# Construction and Validation of a Predictive Model for the Risk of Ventilator-Associated Pneumonia in Elderly ICU Patients

**DOI:** 10.1155/2023/7665184

**Published:** 2023-01-12

**Authors:** Shuhua Li, Linping Shang, Lirong Yuan, Wei Li, Hongyun Kang, Wenting Zhao, Xiaojuan Han, Danxia Su

**Affiliations:** ^1^Nursing College, Shanxi Medical University, Taiyuan, Shanxi, China; ^2^NHC Key Laboratory of Pneumoconiosis, Department of Pulmonary and Critical Care Medicine, The First Hospital of Shanxi Medical University, Taiyuan, Shanxi, China; ^3^Infection Management Department, The First Hospital of Shanxi Medical University, Taiyuan, Shanxi, China

## Abstract

**Background:**

Ventilator-associated pneumonia (VAP) is among the most important hospital-acquired infections in an intensive-care unit setting. However, clinical practice lacks effective theoretical tools for preventing VAP in the elderly.

**Aim:**

To describe the independent factors associated with VAP in elderly intensive-care unit (ICU) patients on mechanical ventilation (MV) and to construct a risk prediction model.

**Methods:**

A total of 1851 elderly patients with MV in ICUs from January 2015 to September 2019 were selected from 12 tertiary hospitals. Study subjects were divided into a model group (*n* = 1219) and a validation group (*n* = 632). Two groups of patients were divided into a VAP group and a non-VAP group and compared. Univariate and logistic regression analyses were used to explore influencing factors for VAP in elderly ICU patients with MV, establish a risk prediction model, and draw a nomogram. We used the area under the receiver operating characteristic curve (AUROC) and the Hosmer–Lemeshow goodness-of-fit test to evaluate the predictive effect of the model. Findings regarding the length of ICU stay, surgery, C-reactive protein (CRP), and the number of reintubations were independent risk factors for VAP in elderly ICU patients with MV. Predictive-model verification results showed that the area under the curve (AUC) of VAP risk after MV in the modeling and verification groups was 0.859 and 0.813 (*P* < 0.001), respectively, while *P* values for the Hosmer–Lemeshow test in these two groups were 0.365 and 0.485, respectively.

**Conclusion:**

The model could effectively predict the occurrence of VAP in elderly patients with MV in ICUs. This study is a retrospective study, so it has not been registered as a clinical study.

## 1. Introduction

Ventilator-associated pneumonia (VAP) is defined as pneumonia that occurs 48 hours after intubation or tracheotomy. Pneumonia that occurs within 48 hours after decanting and extubation from mechanical ventilation (MV) is also included in VAP [1]. VAP prolongs MV time and hospital stay and increases medical costs. VAP also results in atelectasis, MV-related lung injury, respiratory tract obstruction, and other complications. The morbidity and mortality of individuals with VAP vary by country, intensive-care unit (ICU) type, and diagnostic criteria. The incidence of VAP reported in European centers is 18.3 cases per 1000 MV days (MVDs) [2], 18.5 cases per 1000 MVDs in low- and middle-income countries, and 9.0 cases per 1000 MVDs in high-income countries such as the United States (U.S.) [3]. A multicenter investigation in China showed that the incidence of VAP in patients with MV was 9.7–48.4%, or 1.3–28.9 per 1000 MVDs, and the fatality rate was 21.2–43.2% [4]. In addition, delayed diagnosis of VAP can impede treatment or promote the overuse of broad-spectrum antimicrobials [5]. A recent cost assessment in the U.S. estimated the attribution cost of VAP to be $40,144 (95% confidence interval [CI], $36,286–44,220) [6].

The number of elderly patients requiring MV is increasing every year. Due to a decline in immune system function, elderly individuals are more prone to opportunistic infections, which increase the possibility of VAP [7]. At present, several risk factors, including age, history of severe chronic obstructive pulmonary disease, admission type, and gender, have been identified as being associated with VAP [8, 9]. However, few studies exist that assess the risk of VAP in elderly ICU patients with MV. Furthermore, models for predicting VAP in these patients are limited. Clinical practice lacks effective theoretical tools for preventing VAP in the elderly.

The VAP prediction nomogram is recognized as a user-friendly prognostic tool that helps clinicians evaluate the risk of VAP and make better clinical decisions [8]. In this study, we used the classic logistic regression method to develop a VAP prediction model and draw a nomogram based on the baseline clinical characteristics of elderly ICU patients with MV to explore the factors influencing the occurrence of VAP and to provide an effective predictive tool for early identification of high-risk patients.

## 2. Methods

### 2.1. Study Population

In this retrospective analysis, the study cohort included elderly patients who were in ICUs with MV from January 2015 to September 2019 in 12 tertiary hospitals in Shanxi Province, China. The inclusion criteria were as follows: (1) ventilator use ≥48 h; (2) no VAP infection prior to MV; and (3) age ≥65 years. The sole exclusion criteria were as follows: (1) pneumonia diagnosed before MV; (2) death ceased treatment, discharged, or transferred within 48 h; and (3) patients with incomplete case data. Patients were diagnosed in accordance with the criteria of the Guidelines for the Diagnosis and Treatment of Chinese Adult Hospital-Acquired Pneumonia and VAP (2018 edition) [1]. We diagnosed VAP when the chest X-ray or computed tomography (CT) between 48 hours after the initiation of MV and discharge showed new or progressive infiltrating shadows, consolidation shadows, or ground-glass shadows with two or more of the following symptoms: (1) fever (body temperature >38°C); (2) purulent discharge from the airway; or (3) peripheral-blood white blood cell (WBC) count >10 × 10^9^/L or <4 × 10^9^/L.

Based on the study inclusion and exclusion criteria, 1219 patients were identified from 9 tertiary hospitals from January 2015 to December 2017 and divided into the VAP and non-VAP groups (137 patients in the VAP group and 1082 in the non-VAP group). 632 elderly ICU patients receiving mechanical ventilation (MV) in 3 other tertiary hospitals from January 2018 to September 2019 were selected as the model validation group using the convenience sampling method. The selection criteria for research subjects and VAP diagnostic criteria were the same. 67 individuals were in the VAP group, and 565 individuals were in the non-VAP group.

### 2.2. Research Tools

A VAP clinical data questionnaire for elderly ICU patients with MV was compiled by researching the literature and convening an expert group meeting. The questionnaire had four parts, covering 27 indicators: (1) clinical demographics of patients, including gender, length of ICU stay, fever, days with fever, smoking, alcohol consumption, admission department, underlying diseases, the reason for admission, VAP prevention, and the composition of the tracheal tube cuff; (2) surgery and antibiotics, including whether or not surgery is performed, the number of surgeries performed, the duration of antibiotics used after surgery, and the use of antibiotics in combination; (3) laboratory indicators such as CRP level, urinary WBC count, and procalcitonin (PCT) level; (4) invasive monitoring conditions, including duration days of CVC, indwelling CVC, number of indwelling CVC, duration days of a urinary catheter, indwelling urinary catheter, number of an indwelling urinary catheter, duration of MV, and number of reintubations.

### 2.3. Data Collection Methods

Through a computer terminal, we used the hospital infection real-time monitoring system to collect clinical data according to the questionnaire we designed. Data were collected by two researchers who worked in the Department of Respiratory Medicine and the Department of Nosocomiology. Qualified respiratory physicians provided the diagnosis of VAP. Data were input and statistically analyzed using two-person unified coding, and then a third party checked all data to ensure accuracy. This study was approved by the Ethics Committee of the First Hospital of Shanxi Medical University (2020KK072). Informed consent was not required because of the study's retrospective nature.

### 2.4. Statistical Methods

SPSS version 20.0 (IBM Corp., Armonk, NY, USA) was used for data analysis. The count data are statistically described by frequency and percentage, and the VAP and non-VAP groups were compared using the chi-square test or Fisher's exact probability method. The Wilcoxon rank-sum test was used for comparisons between more than two groups. We included variables with *P* < 0.05 in the univariate analysis in our logistic regression analysis and used the forward stepwise method to determine which variables to include in the predictive model. *P* < 0.05 indicated that the differences were statistically significant. Multicollinearity between independent variables was determined by calculating the variance inflation factor (VIF). Finally, according to the corresponding partial regression coefficient of each variable, we constructed an equation to establish the predictive model of VAP in elderly ICU patients with MV and visualized patient risk using a line graph. The area under the receiver operating characteristic curve (AUROC) and the Hosmer–Lemeshow goodness-of-fit test were used to evaluate the effect of the predictive model.

## 3. Results

### 3.1. Incidence of VAP

From the initial modeling cohort of 1307 elderly patients on MV in the ICU, 88 had incomplete data and were excluded for not meeting the inclusion criteria. Thus, the final modeling cohort consisted of 1219 patients, of whom 137 (11.24%) developed VAP after MV.

### 3.2. Univariate-Regression Analysis

To facilitate statistical analysis and clinical application, we grouped continuous variables, such as length of ICU stay, days with fever, the number of surgeries, duration of antibiotics used after surgery, days and number of CVCs, days and number of urinary catheters and duration of MV days, and number of reintubations by the median. Single-factor analysis revealed 15 statistically significant factors: length of ICU stay, surgery, number of surgeries, fever, days with fever, COPD, CRP, PCT, urinary WBC count, indwelling central-venous catheter, duration of CVC days, number of indwelling CVC, number of indwelling urinary catheter, indwelling urinary catheter, and number of reintubations (all *P* < 0.05; Table 1).

### 3.3. Multivariate-Regression Analysis

We included factors with statistical significance in univariate analysis in dichotomous nonconditional logistic regression analysis and used the stepwise forward method and likelihood ratio test to further screen the influencing factors. The independent-variable assignment was as follows: length of ICU stay: ≤19 days = 0, >19 days = 1; surgery: no = 0, yes = 1; number of surgeries: ≤2 = 0, >2 = 1; fever: no = 0, yes = 1; days with fever: ≤2 = 0, >2 = 1; combined with COPD: no = 0, yes = 1; CRP: ≤8 mg/L = 0, >8 mg/L = 1; PCT: ≤0.25 ng/mL = 0, >0.25 ng/mL = 1; urine WBC count: normal = 0, abnormal = 1; indwelling central-venous catheter: no = 0, yes = 1; duration days of CVC: ≤7 = 0, >7 = 1; number of CVCs: ≤2 = 0, >2 = 1; Indwelling urinary catheter: no = 0, yes = 1; number of indwelling urinary catheter: ≤2 = 0, >2 = 1; number of reintubations: ≤2 = 0, >2 = 1. The results showed that length of hospital stay, surgery, COPD, CRP, PCT, and the number of reintubations were factors that independently influenced the occurrence of VAP in elderly ICU patients with MV (Table 2). We diagnosed the collinearity of these six factors. Their VIFs were, respectively, 1.1, 1.1, 1.1, 1.0, 1.1, and 1.1—all <10, indicating that these factors did not have a multicollinearity problem.

### 3.4. Construction of VAP Risk Prediction Model

Based on the above six independent influencing factors and their corresponding regression coefficients, a predictive model of VAP occurrence in elderly ICU patients with MV was established:

*Z* = −4.231 + 0.473 × (length of ICU days) + 1.937 × (surgery) − 1.950 × (COPD) + 1.092 × (CRP) − 0.663 × (PCT) + 0.839 × (number of reintubations).

**Figure 1:**
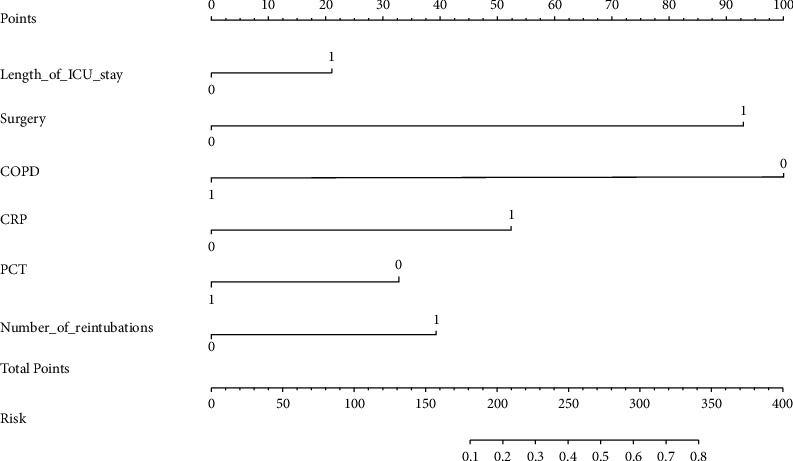
VAP risk prediction nomogram of elderly ICU patients on mechanical ventilation.

A nomogram of VAP in these patients was constructed (Figure 1). Scores corresponding to each indicator can be obtained from the classification of variables in the nomogram, and these scores are added together to calculate the total score. In this study, the predicted probability corresponding to the total score was the probability of VAP occurrence in elderly ICU patients on MV.

### 3.5. Validation of Predictive Models

**Figure 2:**
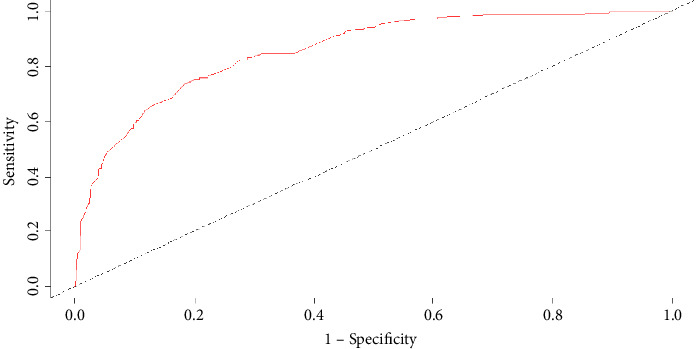
ROC curve of the prediction model in the modeling group.

**Figure 3:**
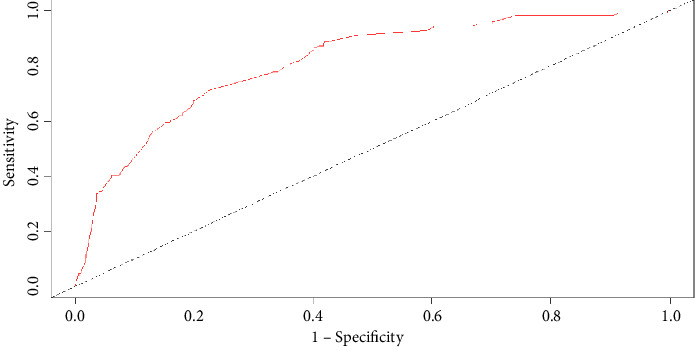
ROC curve of the prediction model in the validation group.

In this study, the differentiation and calibration of the predictive model were evaluated using ROC curve analysis and the Hosmer–Lemeshow test. The results showed that the AUC of VAP risk after intubation in the modeling group was 0.859 (95% CI: 0.828–0.890; *P* < 0.001; Figure 2). Sensitivity, specificity, and the Youden index were 0.723, 0.848, and 0.570, respectively. The AUC for the risk of pulmonary infection after intubation in the validation group was 0.813 (95% CI: 0.700–0.850; *P* < 0.001; Figure 3). Sensitivity, specificity, and the Youden index were 0.714, 0.806, and 0.504, respectively. The Hosmer–Lemeshow test showed *P* values for the modeling and validation groups of 0.365 (*χ*^2^ = 8.734) and 0.485 (*χ*^2^ = 7.484), respectively.

## 4. Discussion

VAP is one of the most common infections in patients with MV [10]. The present study found that the incidence of VAP in elderly MV patients in the ICU setting was 11.24%, which was close to that in elderly patients in other geographic locations (10%) [11] but lower than that found in Europe, where the rates were 16.6% for 65–74 years old and 13% for ≥75 years old [12]. The reasons for the difference from the present study might be that the European study did not reclassify patients >65 years old and that the diagnostic criteria and definitions of VAP differ between countries. The present study also determined that length of stay, surgery, CRP, and several uses of a ventilator were independent risk factors for VAP in elderly ICU patients with MV, while PCT and COPD were protective factors against VAP in this cohort.

Previous studies showed that VAP occurred in 27% of ICU patients receiving MV [11, 13]. Pneumonia is associated with a prolonged hospital stay. The total hospital stay of patients with postoperative pneumonia is 4.7 days longer than average; that of patients with tumors is 5.1 days longer; and that of patients with cerebrovascular disease is 4.0 days longer [9]. We found that prolonged ICU hospitalization was a risk factor for VAP (odds ratio (OR) = 1.542), possibly because VAP was associated with longer MV duration, thereby increasing the length of ICU stay and medical costs [14]. These new and published data suggest that to reduce costs, it is imperative to identify the risk factors associated with VAP and initiate preventive measures promptly.

The incidence of VAP in trauma patients is 17.8% [15], which, in part, may be explained by changes in immune function after major trauma [2]. Elderly patients generally have low immune function and complications from a variety of chronic diseases, making them susceptible to VAP. Surgery may lead to decreased lung capacity, failure to clear secretions, and a reduced cough [16], all of which increase the risk of VAP. We found that surgery was highly positively correlated with the risk of VAP in elderly patients with MV in ICUs (odds ratio (OR) = 6.937) and that the OR value of surgery was significantly higher than those of other influencing factors. Together, this suggests that surgery was the most important influencing factor in the occurrence of VAP. Considering these findings, it seems reasonable for clinical staff to provide additional attention to patients undergoing surgery and administer personalized VAP prevention and control measures to these individuals. In addition, the specific type of surgery may affect the incidence of VAP. The types of surgery included in this study were mainly traumatic brain injury and cardiac surgery. The results showed that the incidence of VAP was 36% in patients with traumatic brain injuries [17]. In patients who had mechanical ventilation after cardiac surgery for more than 48 hours, the average incidence of VAP was 35.2% (range, 17.9%–53%) [18]. Subsequent studies will further analyze the incidence and influencing factors of VAP in elderly patients with different types of surgery.

PCT and CRP are commonly used clinical markers of infection. Interestingly, CRP increased by 86.22% in patients with COVID-19 [19]. Predictive models for severe COVID-19 showed that CRP was associated with a higher chance of severe disease [20, 21]. Recently, the combination of PCT and CRP was employed in the diagnosis of VAP. The combination was found effective in the diagnosis of VAP and in tracking the treatment effects. Additionally, dynamic monitoring of PCT and CRP predicted the prognosis of VAP patients [22] and is consistent with the results of the present study. Differences in VAP condition, pathogen type, and detection time lead to changes in the levels of inflammatory markers such as PCT and CRP. Yet, such changes may not be synchronous with the disease. Unexpectedly, we found CRP to be a risk factor and PCT to be a protective one for VAP. Due to the limitations of retrospective data analysis, this study could not determine the correlation between the time and timing of the blood specimen examination and the incidence of VAP. In future work, the relationship between the occurrence of VAP and the time and value of blood specimen collection should be further studied.

Reintubation is an important predictor of VAP [23]. The incidence of VAP in patients who were reintubated more than twice (28.4%) was higher than that in patients reintubated ≤2 times (16.2%), [7]. This is similar to the results of the present study. This may be secondary to the loss of upper respiratory tract filtration and humidification and the loss of the tracheal cough reflex, all of which promote colonization of the trachea by pathogenic microorganisms. Colonized pathogens form biofilms and increase the likelihood of lower respiratory tract infection [24, 25]. It is recommended that clinical staff employ the spontaneous breathing test and balloon leakage test prior to extubation to minimize reintubation rates.

Despite recent advances in microbial technology, the epidemiological data and diagnostic criteria of VAP remain controversial, complicating the interpretation of treatment, prevention, and outcome studies [10]. The increased incidence observed in COPD patients might be due to prolonged invasive MV (muscle weakness), a high incidence of microinhalation and bacterial colonization (defective mucosal ciliary clearance), and changes in local and general host defense mechanisms [26]. COPD is a risk factor for pneumonia after craniotomy [9]. In contrast, we found that COPD was a protective factor against the occurrence of VAP. The discrepancy might be secondary to the selection of study subjects and the classification of disease severity. The low number of COPD patients with VAP included in this study might also be related to the low rate of pulmonary function examination and the high rate of missed diagnosis in China [27].

In this study, we established a risk prediction model for elderly ICU patients with MV. The results showed that the AUC of the prediction model in the modeling group was 0.859 and that in the verification group was 0.813, a decrease of only 0.046. This indicated that the risk prediction model had a strong ability and high accuracy in predicting the risk of VAP in elderly patients with MV in the ICU. In addition, the calibration degree of the prediction model was good. More specifically, in the calibration degree test, the *P* values for the modeling and verification groups were 0.565 and 0.370, respectively. This indicated that the probability of VAP risk predicted by the model was close to the actual incidence. For simplicity, we constructed a nomogram based on the model. The nomogram is easy to understand and can be used quickly. Medical personnel can use this model after the MV of patients to perform dynamic risk assessment and screen the high-risk population, allowing for effective preventive measures to achieve early prediction, early prevention, and early intervention.

Similar studies have been carried out in the past but in older patients (2011–2015) [7]. We added in newer data, and expanding it to ≥65 does add new data to the literature. There are several limitations to this study. First, this is a multicenter, retrospective study, and thus information on specific types of parameter settings and sedation while individuals were on the MV is lacking. VAP is known to lead to severe lung injury, and accumulating evidence has suggested that driving pressure and mechanical power could reflect lung injury and alveolar damage [28, 29]. Being limited by the data collected from the information system, our study could not account for the impact of different markers of respiratory mechanics on VAP. Furthermore, the nature of the endotracheal tube, and especially the tube cuff, might have played a role in the results [30]. Although we collected data on tube materials, most hospitals use lower-priced polyvinylchloride tubes due to the influence of national policy to reduce the consumable ratio. Therefore, the influence of tube material on VAP needs further research. In addition, the endpoint of this study was discharge from the hospital. Some patients can develop lung infections after discharge; the incidence of VAP reported in this study might be lower than the true incidence. In terms of model verification, although we adopted a rigorous external verification method, data were collected from only three centers. In the future, the developed model will be applied clinically at multiple centers to refine its accuracy.

## 5. Conclusion

Analysis of over one thousand elderly ICU MV subjects found that >19 days in the ICU, surgery, CRP >8 mg/L, and >2 times of reintubations were associated with increased incidence of VAP. A VAP predictive nomogram, calculated AUC, and the Hosmer–Lemeshow goodness-of-fit test demonstrated acceptable model fit and relatively good performance. In clinical practice, physicians can use our nomogram to assess the risk of VAP development in elderly ICU patients with MV and initiate early preventive strategies. Effective preventive treatment might confer a better prognosis in these critically ill patients.

## Figures and Tables

**Table 1:** Univariate analysis of VAP risk in elderly ICU patients on MV (*n* = 1219, *n* (%)).

		VAP (%)		Total (%)	*χ* ^2^	*P*
		Yes	No			
Gender	Male	91 (66.4)	712 (65.8)	803 (65.9)	0.021	0.885
	Female	46 (33.6)	370 (34.2)	416 (34.1)		

Length of ICU stay	0–19	44 (32.1)	589 (54.4)	633 (51.9)	24.267	<0.001
	>19	93 (67.9)	493 (45.6)	586 (8.1)		

Fever	Yes	103 (75.2)	618 (57.1)	721 (59.1)	16.425	<0.001
	No	34 (24.8)	464 (42.9)	498 (40.9)		

Days with fever	0–2	45 (32.8)	584 (54.0)	629 (51.6)	21.734	<0.001
	>2	92 (67.2)	498 (46.0)	590 (48.4)		

Smoking	Yes	65 (47.4)	440 (40.7)	505 (41.4)	2.304	0.141
	No	72 (52.6)	642 (59.3)	714 (58.6)		

Alcohol consumption	Yes	42 (47.4)	383 (40.7)	505 (41.4)	1.203	0.296
	No	95 (52.6)	699 (59.3)	714 (58.6)		

Admission department	Emergency	8 (5.8)	54 (5.0)	62 (5.1)	0.904	0.636
	Surgical	67 (48.9)	493 (45.6)	560 (45.9)		
	Medical	62 (45.3)	535 (49.4)	597 (49.0)		

Underlying diseases	COPD	2 (1.5)	242 (22.4)	244 (20.0)	33.197	<0.001
	Heart failure	13 (9.5)	112 (10.4)	125 (10.3)	0.098	0.881
	Chronic renal failure	5 (3.6)	25 (2.3)	30 (2.5)	0.908	0.372
	Diabetes mellitus	17 (12.4)	158 (14.6)	175 (14.4)	0.476	0.605
	Cancer	14 (10.2)	135 (12.5)	149 (12.2)	0.578	0.579
	Immune suppression	15 (10.9)	126 (11.6)	141 (11.6)	0.058	0.888

Reason of admission	Respiratory	51 (37.2)	455 (42.1)	506 (41.5)	1.130	0.288
	Cardiovascular	9 (6.6)	79 (7.3)	88 (7.2)	0.097	0.755
	Digestive	8 (5.8)	48 (4.4)	56 (4.6)	0.546	0.460
	Trauma	32 (23.4)	309 (28.6)	341 (28.0)	1.632	0.201
	Severe sepsis	18 (13.1)	114 (10.5)	132 (10.8)	0.853	0.356
	Other	23 (16.8)	137 (12.7)	160 (13.1)	1.816	0.178

VAP prevention	Bed head elevation > 30°	124 (90.5)	952 (88.0)	1076 (88.3)	0.749	0.387
	Daily wake up	94 (68.6)	757 (70.0)	851 (69.8)	0.105	0.746
	DVT prevention	107 (78.1)	865 (79.9)	972 (79.7)	0.255	0.613
	Oral care with chlorhexidine	105 (76.6)	806 (74.5)	911 (74.7)	0.298	0.585
	Tube cuff pressure monitoring >4 times per day	89 (65.0)	687 (63.5)	776 (63.7)	0.114	0.736
	Subglottic drainage	27 (19.7)	194 (17.9)	221 (18.1)	0.259	0.611

Materials of tube	Polyvinylchloride	132 (96.4)	1062 (98.2)	1194 (98.0)	1.964	0.189
	Polyurethane	5 (3.6)	20 (1.8)	25 (2.0)		

Surgery	Yes	87 (63.5)	141 (13.0)	228 (18.7)	203.727	<0.001
	No	50 (36.5)	941 (87.0)	991 (81.3)		

Number of surgeries	0–2	127 (92.7)	1060 (98.0)	1187 (97.4)	—	0.002^*∗*^
	>2	10 (7.3)	22 (2.0)	32 (2.6)		

Antibiotics use after surgery	Yes	132 (96.4)	1047 (96.8)	1179 (96.7)	0.066	0.797
	No	5 (3.6)	35 (3.2)	40 (3.3)		

Duration of antibiotics use after surgery	0–14	71 (51.8)	568 (52.5)	639 (52.4)	0.022	0.882
	>14	66 (48.2)	514 (47.5)	580 (47.6)		

Combined application of antibiotics	Yes	115 (83.9)	930 (86.0)	1045 (85.7)	0.402	0.526
	No	22 (16.1)	152 (14.0)	174 (14.3)		

CRP (mg/L)	>8	78 (56.9)	346 (32.0)	424 (34.8)	33.388	<0.001
	0–8	59 (43.1)	736 (68.0)	795 (65.2)		

PCT (ng/mL)	>0.25	33 (24.1)	357 (33.0)	390 (32.0)	4.434	0.035
	0–0.25	104 (75.9)	725 (67.0)	829 (68.0)		

Urinary WBC count	Abnormal	33 (24.1)	373 (34.5)	406 (33.3)	5.905	0.015
	Normal	401 (75.9)	709 (65.5)	813 (66.7)		

Duration days of CVC	0–7	34 (24.9)	591 (54.6)	625 (51.3)	42.234	<0.001
	>7	103 (75.2)	491 (45.4)	594 (48.7)		

Indwelling CVC	Yes	122 (89.1)	629 (58.1)	751 (61.6)	49.146	0.035
	No	15 (10.9)	453 (41.9)	468 (38.4)		

Number of indwelling CVC	0–2	128 (93.4)	1055 (97.5)	1183 (97.0)	5.905	0.015
	>2	9 (6.6)	27 (2.5)	36 (3.0)		

Duration days of urinary catheter	0–10	65 (47.4)	559 (51.7)	624 (51.2)	0.866	0.352
	>10	72 (52.6)	523 (48.3)	595 (48.8)		

Indwelling urinary catheter	Yes	121 (88.3)	751 (69.4)	872 (71.5)	21.360	<0.001
	No	16 (11.7)	331 (30.6)	347 (28.5)		

Number of indwelling urinary catheter	0–2	115 (83.9)	970 (89.6)	1085 (89.0)	5.905	0.015
	>2	22 (16.1)	112 (10.4)	134 (11.0)		

Duration days of MV	0–10	72 (52.6)	561 (51.8)	633 (51.9)	0.024	0.876
	>10	65 (47.4)	521 (48.2)	586 (48.1)		

Number of reintubations	0–2	123 (89.8)	1027 (94.9)	1150 (94.3)	6.006	0.014
	>2	14 (10.2)	55 (5.1)	69 (5.7)		

Total	137 (11.2)	1082 (88.8)	1219 (100.0)			

^*∗*^Fisher's exact probability method. VAP, ventilator-associated pneumonia; ICU, intensive-care unit; MV, mechanical ventilation; COPD, chronic obstructive pulmonary disease; DVT, deep venous thrombi; CRP, C-reactive protein; PCT, procalcitonin; WBC, white blood cell; CVC, central-venous catheter. Normal WBC counts in urine are 1–10 in males and 0–5 in females.

**Table 2:** Multivariate analysis of VAP risk in elderly ICU patients on MV.

Risk factors	*b*	Wald	*P*	OR	95% CI	
					Lower limit	Upper limit
Length of ICU stay	0.473	3.984	0.046	1.604	1.009	2.551
Surgery	1.937	71.815	<0.001	6.937	4.432	10.857
COPD	−1.950	6.929	0.008	0.142	0.033	0.608
CRP	1.092	24.658	<0.001	2.981	1.937	4.587
PCT	−0.663	7.156	0.007	0.515	0.317	0.837
Number of reintubations	0.839	3.921	0.048	2.313	1.009	5.350
Constant	−4.231	76.976	<0.001	0.015	—	—

VAP, ventilator-associated pneumonia; ICU, intensive-care unit; MV, mechanical ventilation; OR, odds ratio; CI, confidence interval; COPD, chronic obstructive pulmonary disease; CRP, C-reactive protein; PCT, procalcitonin.

## Data Availability

The data that support the findings of this study are available from the corresponding author (shanglp2002@163.com), upon reasonable request.

## Abbreviations

ICU: Intensive care unit
VAP: Ventilator-associated pneumonia
MV: Mechanical ventilation
COPD: Chronic obstructive pulmonary disease
CRP: C-reactive protein
PCT: Procalcitonin
WBC: White blood cell
CVC: Central vein catheterization
CT: Computed tomography
DVT: Deep venous thrombi
AUROC: The area under the receiver operating characteristic curve
AUC: The area under the curve.

## References

[1] Infectology Group (2018). Guidelines for the diagnosis and treatment of hospital-acquired pneumonia and VAP in Chinese adult hospitals (2018 edition)[J]. *Chinese Journal of Tuberculosis and Respiratory Medicine*.

[2] Koulenti D., Tsigou E., Rello J. (2017). Nosocomial pneumonia in 27 ICUs in Europe: perspectives from the EU-VAP/CAP study. *European Journal of Clinical Microbiology & Infectious Diseases*.

[3] Bonell A., Azarrafiy R., Huong V. T. L. (2019). A systematic review and meta-analysis of ventilator-associated pneumonia in adults in Asia: an analysis of national income level on incidence and etiology. *Clinical Infectious Diseases*.

[4] Zhang J., He Q., Zhou Bo, Deng X. (2015). Analysis of risk factors for VAP in ICU patients[J]. *Chinese Journal of Nosocomial Infection*.

[5] Frondelius T., Atkova I., Miettunen J., Rello J., Jansson M. M (2022). Diagnostic and prognostic prediction models in ventilator-associated pneumonia: systematic review and meta-analysis of prediction modelling studies. *Journal of Critical Care*.

[6] Zimlichman E., Henderson D., Tamir O. (2013). Healthcare-associated infections: a meta-analysis of costs and financial impact on the US health care system. *JAMA Internal Medicine*.

[7] Xu Y., Lai C., Xu G. (2019). Risk factors of ventilator-associated pneumonia in elderly patients receiving mechanical ventilation. *Clinical Interventions in Aging*.

[8] Wu Z., Liu Y., Xu J. (2020). A ventilator-associated pneumonia prediction model in patients with acute respiratory distress syndrome. *Clinical Infectious Diseases*.

[9] Zhang D., Zhuo H., Yang G. (2020). Postoperative pneumonia after craniotomy: incidence, risk factors and prediction with a nomogram. *Journal of Hospital Infection*.

[10] Papazian L., Klompas M., Luyt C. E. (2020). Ventilator-associated pneumonia in adults: a narrative review. *Intensive Care Medicine*.

[11] Metersky M. L., Wang Y., Klompas M., Eckenrode S., Bakullari A., Eldridge N. (2016). Trend in ventilator-associated pneumonia rates between 2005 and 2013. *JAMA*.

[12] Blot S., Koulenti D., Dimopoulos G. (2014). Prevalence, risk factors, and mortality for ventilator-associated pneumonia in middle-aged, old, and very old critically ill patients. *Critical Care Medicine*.

[13] Kalanuria A. A., Mirski M., Zai W., Mirski M. (2014). Ventilator-associated pneumonia in the ICU. *Critical Care*.

[14] Vincent J. L., de Souza Barros D., Cianferoni S. (2010). Diagnosis, management and prevention of ventilator-associated pneumonia: an update. *Drugs*.

[15] Cook A., Norwood S., Berne J. (2010). Ventilator-associated pneumonia is more common and of less consequence in trauma patients compared with other critically ill patients. *The Journal of Trauma, Injury, Infection, and Critical Care*.

[16] Sligl W. I., Eurich D. T., Marrie T. J., Majumdar S. R. (2011). Only severely limited, premorbid functional status is associated with short- and long-term mortality in patients with pneumonia who are critically ill: a prospective observational study. *Chest*.

[17] Li Y., Liu C., Xiao W., Song T., Wang S. (2020). Incidence, risk factors, and outcomes of ventilator-associated pneumonia in traumatic brain injury: a meta-analysis. *Neurocritical Care*.

[18] He S., Chen B., Li W. (2014). Ventilator-associated pneumonia after cardiac surgery: a meta-analysis and systematic review. *The Journal of Thoracic and Cardiovascular Surgery*.

[19] Li R., Tian J., Yang F. (2020). Clinical characteristics of 225 patients with COVID‐19 in a tertiary hospital near Wuhan, China. *Journal of Clinical Virology*.

[20] Gong J., Ou J., Qiu X. (2020). A tool to early predict severe coronavirus disease 2019 (COVID-19): a multicenter study using the risk nomogram in Wuhan and Guangdong, China. *Clinical Infectious Diseases*.

[21] Yao Z., Zheng X., Zheng Z., Wu K., Zheng J. (2021). Construction and validation of a machine learning-based nomogram: a tool to predict the risk of getting severe coronavirus disease 2019 (COVID-19). *Immunity, Inflammation, and Disease*.

[22] Isguder R., Ceylan G., Agin H., Gülfidan G., Ayhan Y., Devrim I (2017). New parameters for childhood ventilator associated pneumonia diagnosis. *Pediatric Pulmonology*.

[23] Nakaviroj S., Cherdrungsi R., Chaiwat O. (2014). Incidence and risk factors for ventilator-associated pneumonia in the surgical intensive care unit, Siriraj Hospital. *Medical Journal of the Medical Association of Thailand*.

[24] Sands K. M., Wilson M. J., Lewis M. A. (2017). Respiratory pathogen colonization of dental plaque, the lower airways, and endotracheal tube biofilms during mechanical ventilation. *Journal of Critical Care*.

[25] Webster T., Machado M. (2016). Decreased *Pseudomonas aeruginosa* biofilm formation on nanomodified endotracheal tubes: a dynamic lung model. *International Journal of Nanomedicine*.

[26] Rouze A., Cottereau A., Nseir S. (2014). Chronic obstructive pulmonary disease and the risk for ventilator-associated pneumonia. *Current Opinion in Critical Care*.

[27] Wang C., Xu J., Yang L. (2018). Prevalence and risk factors of chronic obstructive pulmonary disease in China (the China Pulmonary Health [CPH] study): a national cross-sectional study. *The Lancet*.

[28] Amato M. B., Meade M. O., Slutsky A. S. (2015). Driving pressure and survival in the acute respiratory distress syndrome. *New England Journal of Medicine*.

[29] Cressoni M., Gotti M., Chiurazzi C. (2016). Mechanical power and development of ventilator-induced lung injury. *Anesthesiology*.

[30] Zanella A., Scaravilli V., Isgrò S. (2011). Fluid leakage across tracheal tube cuff, effect of different cuff material, shape, and positive expiratory pressure: a bench-top study. *Intensive Care Medicine*.

